# Type I Interferon α/β Receptor-Mediated Signaling Negatively Regulates Antiviral Cytokine Responses in Murine Bone-Marrow-Derived Mast Cells and Protects the Cells from Virus-Induced Cell Death

**DOI:** 10.3390/ijms21239041

**Published:** 2020-11-27

**Authors:** Maedeh Darzianiazizi, Yeganeh Mehrani, Lily Chan, Robert C. Mould, Raveendra R. Kulkarni, Shayan Sharif, Byram W. Bridle, Khalil Karimi

**Affiliations:** 1 Department of Pathobiology, Ontario Veterinary College, University of Guelph, Guelph, ON N1G 2W1, Canada; mahi.azizi@uoguelph.ca (M.D.); ymehrani@uoguelph.ca (Y.M.); lchan12@uoguelph.ca (L.C.); rmould@uoguelph.ca (R.C.M.); shayan@uoguelph.ca (S.S.); 2 Department of Population Health and Pathobiology, College of Veterinary Medicine, North Carolina State University, Raleigh, NC 27607, USA; rrkulkar@ncsu.edu

**Keywords:** bone-marrow-derived mast cells, mast cells, vesicular stomatitis virus, cytokine response, type I interferon, flow cytometry

## Abstract

Mast cells (MCs) are critical for initiating inflammatory responses to pathogens including viruses. Type I interferons (IFNs) that exert their antiviral functions by interacting with the type I IFN receptor (IFNAR) play a central role in host cellular responses to viruses. Given that virus-induced excessive toxic inflammatory responses are associated with aberrant IFNAR signaling and considering MCs are an early source of inflammatory cytokines during viral infections, we sought to determine whether IFNAR signaling plays a role in antiviral cytokine responses of MCs. IFNAR-intact, IFNAR-blocked, and IFNAR-knockout (IFNAR^−/−^) bone-marrow-derived MCs (BMMCs) were treated in vitro with a recombinant vesicular stomatitis virus (rVSVΔm51) to assess cytokine production by these cells. All groups of MCs produced the cytokines interleukin-6 and tumor necrosis factor-α in response to rVSVΔm51. However, production of the cytokines was lowest in IFNAR-intact cells as compared with IFNAR^−/−^ or IFNAR-blocked cells at 20 h post-stimulation. Surprisingly, rVSVΔm51 was capable of infecting BMMCs, but functional IFNAR signaling was able to protect these cells from virus-induced death. This study showed that BMMCs produced pro-inflammatory cytokines in response to rVSVΔm51 and that IFNAR signaling was required to down-modulate these responses and protect the cells from dying from viral infection.

## 1. Introduction

The biological role of mast cells (MCs) was initially attributed to allergic inflammation [1,2]. However, as time passed, research findings highlighted MCs as important sentinel cells in tissues for priming and coordinating host defenses to various pathogens, including viruses [3]. Widely spread throughout the body, MCs mainly reside in tissues interfacing with the external environment, like skin, intestines, airways, and the genitourinary tract [4,5,6,7]. Such strategic locations enable MCs to promptly respond to pathogens, allergens, or other intrusions. Vesicular stomatitis caused by vesicular stomatitis virus (VSV) is a disease of livestock, and the virus can infect humans too [8]. Vesicular stomatitis occurs seasonally in the Southeastern USA, Southern Mexico, throughout Central America, and in Northern South America, and emerges from tropical areas to cause sporadic epidemics in cooler climates during the summer months. Interestingly, recombinant VSV is also used as a vaccine platform [9] and an oncolytic agent [10], but its capacity to induce mucosal immune responses has not yet been fully elucidated. MCs have developed a wide range of mechanisms to deal with potential dangers. These cells can be activated via complement receptors, crosslinking of immunoglobulin-E bound to high-affinity receptors for the constant fragment region of immunoglobulin-E, and recognition of pathogen-associated molecular patterns via pattern recognition receptors [11]. Activation of MCs leads to degranulation and release of granular components, including a variety of pre-formed inflammatory mediators such as histamine, heparin, proteases, chymase, antimicrobial peptides, and tumor necrosis factor (TNF)-α. Shortly after degranulation, de novo synthesis of other mediators begins, including leukotriene-C4 and prostaglandin-D2 [12,13]. Finally, over the course of hours, a wide range of inflammatory cytokines and chemokines are produced. MC-derived inflammatory mediators enhance mobilization of other effector leukocytes to the original site of the insult [14]. The nature of the stimulus has been reported to influence the nature of the response of MCs. For example, recognition of *Escherichia coli* adhesion fimbriae by CD48 leads to the release of TNF-α by murine bone-marrow-derived MCs (BMMCs) [15], whereas sensing fungal β-glucan by Dectin-1 causes the release of leukotriene-C4 by human MCs [16]. Furthermore, various Toll-like receptor (TLR) ligands induce inflammatory cytokines and chemokines in BMMCs [17,18,19,20,21], of which TLR2- and TLR4-mediated inflammatory responses have been reported to occur independently of degranulation [19,21,22,23]. Induction of antiviral cytokines and chemokines by MCs appears to occur via retinoic acid-inducible gene I/mitochondrial antiviral signaling protein/melanoma differentiation-associated protein 5-mediated recognition of viral products leading to the induction of IFN-α and -β, and chemokines [24,25,26,27], independently of classical MC degranulation [24,28,29,30,31,32]. Human MCs have also been shown to selectively induce certain types of inflammatory cytokines and chemokines in response to treatment with IFN-α_2_ and IFN-γ independently of degranulation [33,34]. An antiviral response by MCs can also occur indirectly through detection of IL-33 produced by infected epithelial cells, leading to induction of TNF-α and interleukin (IL)-6 without degranulation [35]. Human MCs induce type I IFNs in response to several viruses, including herpes simplex virus [35], respiratory syncytial virus [36,37], reovirus [36,38,39], influenza virus [36], dengue virus [25,26,40], Newcastle disease virus [20], hantavirus [41], and Sendai virus [42]. It has also been shown that IFNs have a profound impact on cytokine and chemokine production by human MCs treated with reovirus and respiratory syncytial virus in vitro [33]. Type I IFNs, which signal through the type I IFN receptor (IFNAR), are crucial for host defense against viruses, and virus-induced impairment of IFNAR signaling is correlated with immune-mediated pathogenesis of a variety of viral infections [43]. While MCs can be protective in some viral infections [35,44], they can also be detrimental due to excessive production of inflammatory mediators that promote immunopathogenesis of viral infections [45]. Given the limited information on the role of type I IFN-mediated signaling on the modulation of antiviral responses of MCs, this was explored in this in vitro study using murine BMMCs.

## 2. Results

### 2.1. BMMCs Could Be Infected by Wild-Type (WT) rVSV

**Figure 1:**
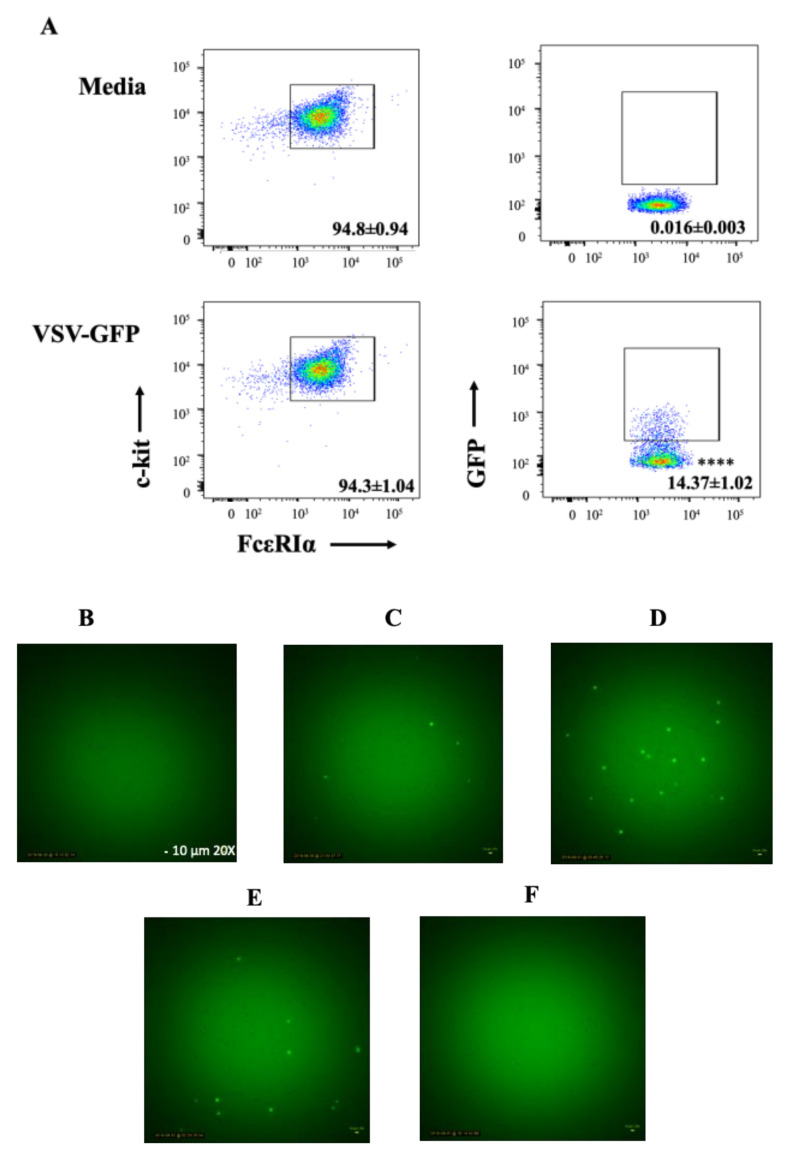
Murine bone-marrow-derived mast cells (BMMCs) were susceptible to infection with wild-type recombinant vesicular stomatitis virus (VSV). BMMCs were left untreated in the cell culture medium or treated with a multiplicity of infection of 10 of wild-type VSV that carried a transgene encoding enhanced green fluorescent protein (GFP). (**A**) Ten hours later, BMMCs were harvested and stained for FCεRIα and c-Kit. GFP ^+^ cells, representative of VSV-infected BMMCs, were detected using flow cytometry. Dot plots are representative of four replicate experiments showing means and standard deviations of GFP^+^FCεRIα^+^c-Kit^+^ BMMCs. (**B**–**F**) Fluorescent microscopy (20×) of VSV-GFP-treated BMMCs (representative of three experimental replicates) at (**B**) 5 min, (**C**) 6 h, (**D**) 10 h, (**E**) 12 h, and (**F**) 24 h after exposure to VSV. Student’s *t*-tests were used to define statistical significance. **** *p* < 0.0001 for GFP^+^ cells in the VSV-GFP-infected versus uninfected cells.

To explore the potential for MCs to be infected with rVSV, BMMCs were initially either treated with medium or WT rVSV encoding a transgene for full-length enhanced green fluorescent protein (GFP) at a multiplicity of infection (MOI) of 10. The reason WT rVSV was used instead of rVSVΔm51 in these experiments was accessibility to GFP-tagged viruses. Flow cytometry data showed that a mean of 14.37% ± 1.02% (standard deviation) of c-kit^+^FcεRIα^+^ BMMCs were positive for GFP expression at 10 h after exposure to the virus (Figure 1A). This suggested that at least some of the BMMCs could be infected with rVSV and support VSV-mediated expression of a transgene. In addition, time-lapse video microscopy of BMMCs confirmed that these cells could be infected by rVSV as demonstrated by them fluorescing green by 10 h post-treatment. BMMCs expressed GFP by six hours after adding WT rVSV-GFP to the cells. Expression of GFP peaked at 10 h and started to diminish at 12 h post-infection. No more GFP-positive cells were visible 24 h after being exposed to the virus (Figure 1B–F). The gradual disappearance of GFP-positive cells implied the possibility of virus-induced cell death.

### 2.2. BMMCs Produced IL-6 and TNF-α in Response to rVSVΔm51

**Figure 2:**
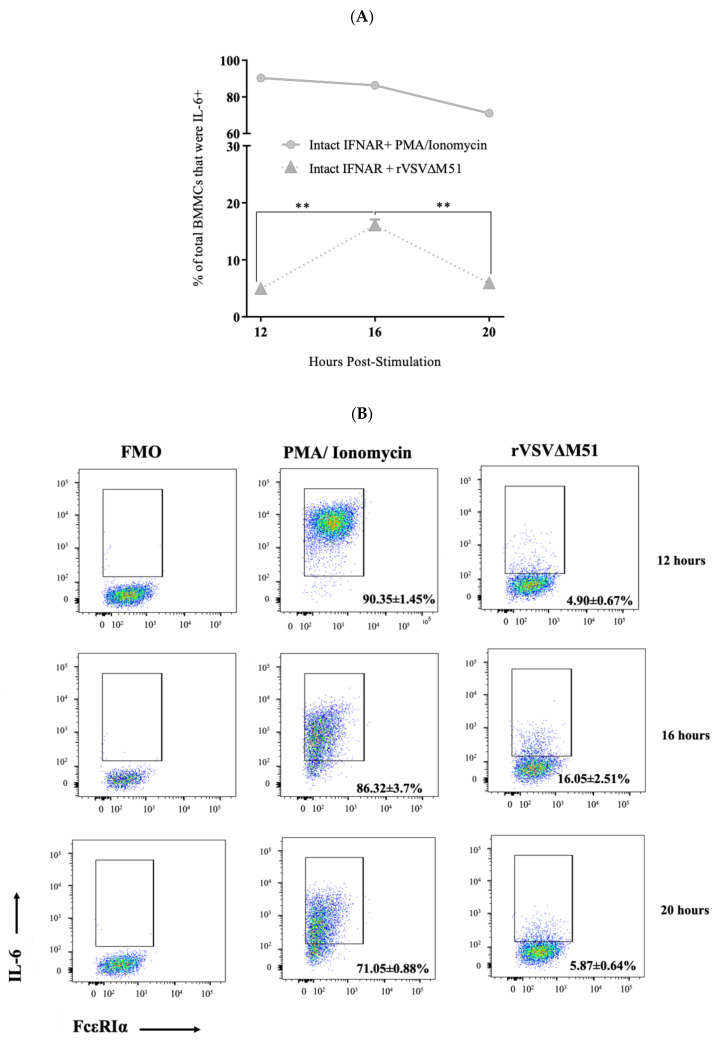

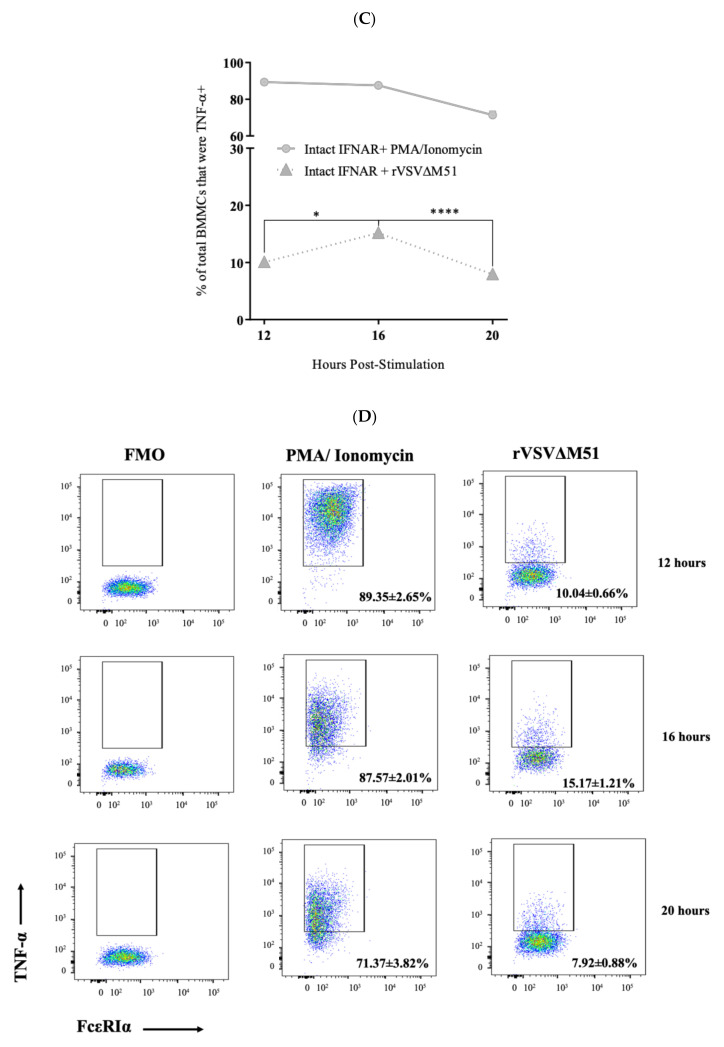
Kinetics of cytokine production by murine bone-marrow-derived mast cells (BMMCs) after infection with a recombinant strain of vesicular stomatitis virus (rVSVΔm51). BMMCs were infected with rVSVΔm51 at a multiplicity of infection of 10. At 12, 16, and 20 h post-infection, the cells were stained for the surface markers FcεRIα and c-kit, as well as intracellular cytokines interleukin (IL)-6 and tumor necrosis factor (TNF)-α. The cells were then analyzed using flow cytometry. Fluorescence minus one (FMA) controls were used to set gates to identify IL-6^+^ and TNF-α^+^ cells. Phorbol 12-myristate 13-acetate (PMA) and ionomycin were used as a positive control stimulus. Graphs and representative dot plots show means and standard deviations pooled from four experimental replicates of (**A**,**B**) IL-6^+^ and (**C**,**D**) TNF-α^+^ FcεRIα^+^ c-Kit^+^ mast cells at 12, 16, and 20 h post-infection. Two-way analysis of variance with Tukey’s multiple comparison test was used to define statistical significance as * *p* < 0.05; ** *p* < 0.001; **** *p* < 0.0001.

BMMCs infected with rVSVΔm51 were assessed for potential production of IL-6 and TNF-α using flow cytometry after intracellular staining. At 16 h post-infection, a mean of 16.05% ± 2.51% (standard deviation) and 15% ± 1.21% of BMMCs were positive for IL-6 and TNF-α, respectively (Figure 2). To study the kinetics of virus-induced cytokine production in BMMCs, the cells were infected with rVSVΔm51 at a MOI of 10. Phorbol 12-myristate 13-acetate (PMA) and ionomycin-mediated stimulation was used as a positive control for detecting intracellular IL-6 and TNF-α. At 12 h post-infection with the virus, IL-6 and TNF-α were detectable in BMMCs (4.90% ± 0.67% and 10.4% ± 0.66%, respectively). The production of IL-6 and TNF-α increased at 16 h (16.05% ± 2.51% and 15.17% ± 1.21%, respectively), but declined at 20 h post-infection (5.87% ± 0.64% and 7.92% ± 0.88%, respectively) (Figure 2). This provided a baseline cytokine profile for rVSVΔm51-treated BMMCs.

### 2.3. Virus-Infected BMMCs Produced Cytokines in a Virus-Dose-Dependent Manner

**Figure 3:**
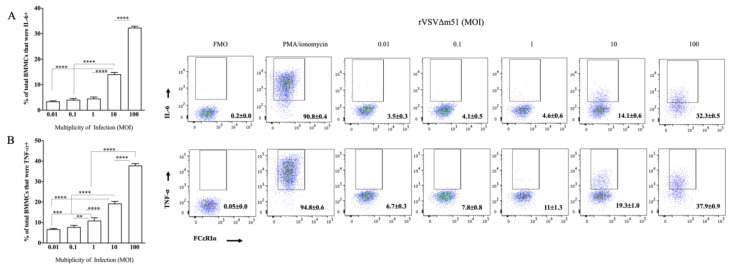
Cytokine responses of murine bone-marrow-derived mast cells (BMMCs) to recombinant vesicular stomatitis virus (rVSVΔm51) were dose-dependent. BMMCs were infected with rVSVΔm51 at a multiplicity of infection (MOI) of 0.01, 0.1, 1, 10, or 100. After 16 h, the cells were stained for the surface markers FcεRIα and c-kit as well as intracellular cytokines interleukin (IL)-6 and tumor necrosis factor (TNF)-α. The cells were then analyzed by flow cytometry, and fluorescence minus one (FMO) controls were used to set gates to identify IL-6^+^ and TNF-α^+^ cells. Graphs and representative dot plots show means and standard deviations pooled from four experimental replicates of (**A**) IL-6 and (**B**) TNF-α expression in FcεRIα^+^c-Kit^+^ mast cells after infection with rVSVΔM51 or treatment with phorbol 12-myristate 13-acetate (PMA) and ionomycin as a positive control stimulus. Two-way analysis of variance with Tukey’s multiple comparison test was used to define statistical significance as ** *p* < 0.001; *** *p* < 0.0005; **** *p* < 0.0001.

To investigate whether rVSVΔm51-induced cytokine responses of MCs could be influenced by the dose of virus, BMMCs were infected with rVSVΔm51 at MOIs of 0.01, 0.1, 1, 10, 1 or 100. Sixteen hours post-infection, the frequencies of IL-6- and TNF-α-positive cells correlated with the dose of the virus (Figure 3). The lowest and highest frequency of IL-6 (7.16% ± 0.62% versus 66.95% ± 1.20%) and TNF-α (13.8% ± 0.78% versus 77.77% ± 1.94%) were detected in cells treated with the lowest (0.01) and highest (100) MOI of virus, respectively. The highest frequency of IL-6- and TNF-α-positive cells was starting to approach cells treated with PMA and ionomycin, with 92.85% ± 1.88% and 94.92% ± 1.46% IL-6- and TNF-α-positive cells, respectively.

### 2.4. Infection of BMMCs with rVSVΔm51 Resulted in Cell Death in a Time- and Virus-Dose-Dependent Manner

**Figure 4:**
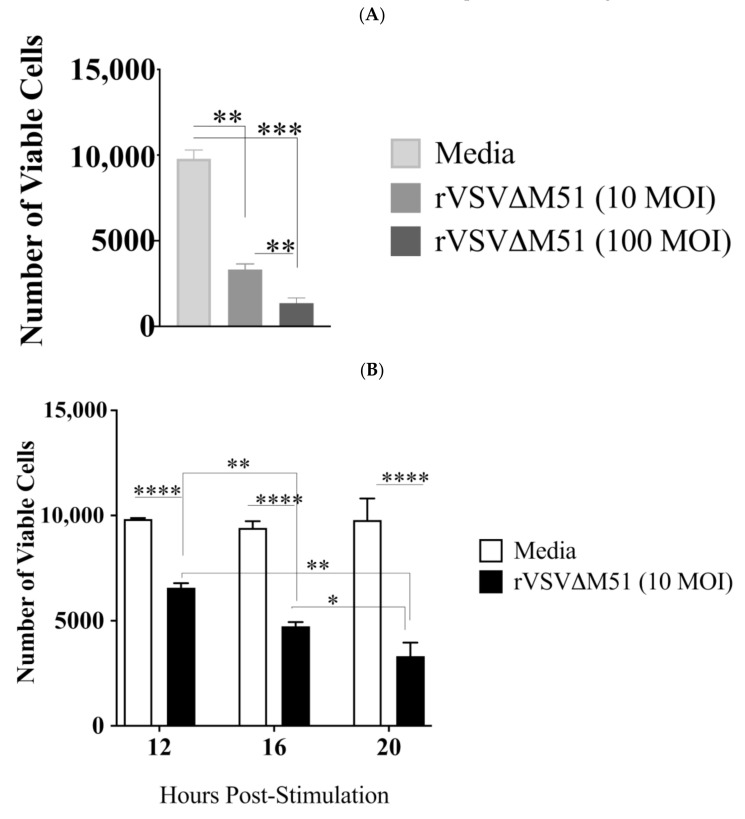
Infection of murine bone-marrow-derived mast cells (BMMCs) with recombinant vesicular stomatitis virus (rVSVΔm51) resulted in cell death. BMMCs were infected with rVSVΔm51 at a multiplicity of infection (MOI) of 10, and at various time points the cells were stained for the surface markers FcεRIα and c-kit. A cell viability dye to discriminate live and dead BMMCs was applied to the cells before flow cytometry analysis. Graphs show means and standard deviations pooled from four experimental replicates of the number of viable cells among total FcεRIα^+^c-Kit^+^ BMMCs at (**A**) 20 h post-infection with 10 or 100 MOI of the virus and (**B**) at 12, 16, and 20 h post-infection with 10 MOI of the virus. Two-way analysis of variance with Tukey’s multiple comparison test was used to define statistical significance as * *p* < 0.05; ** *p* < 0.001; *** *p* < 0.0005; **** *p* < 0.0001.

To study the potential effect of virus-induced cytokine responses on viability of cells, equal numbers of BMMCs were treated with medium or rVSVΔm51 at a MOI of 10. After 12, 16, and 20 h, the cells were stained with a fixable viability dye so that the number of viable cells could be observed using flow cytometry. In contrast to medium-treated cells, cell viability in virus-treated cells showed a decreasing trend over the course of infection with rVSVΔm51. There was a gradual but significant reduction in the number of viable cells between 12 and 20 h post-infection as compared with controls (6572 ± 220.52 cells versus 9834.5 ± 47.9 cells at 12 h; 4,741.75 ± 193.91 cells versus 9408.75 ± 318.48 cells at 16 h; and 3316.25 ± 645.55 cells versus 9781.5 ± 1023.995 cells at 20 h, respectively) (Figure 4B). Furthermore, cell viability had an inverse relationship with the dose of the virus. BMMCs infected with a MOI of 100 of rVSVΔm51 had the lowest number of viable cells (1361.75 ± 577.54) compared with cells infected with a MOI of 10 (3316.25 ± 645.55) at 20 h post-infection (Figure 4A).

### 2.5. IFNAR Signalling Facilitated Down-Regulation of Cytokine Production by BMMCs

**Figure 5:**
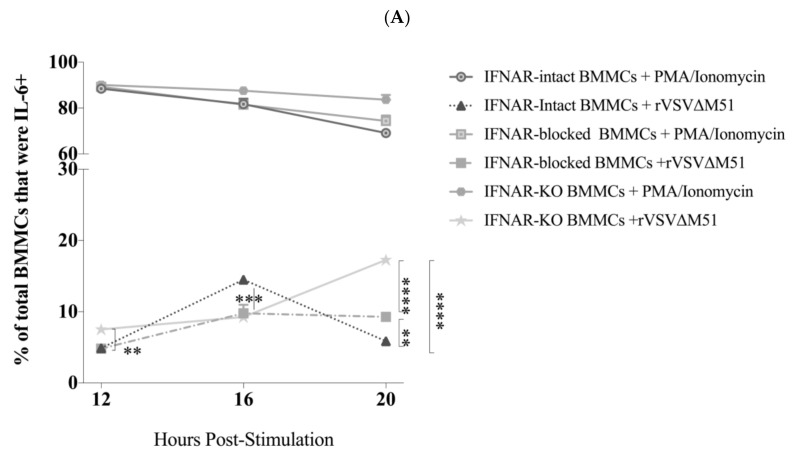

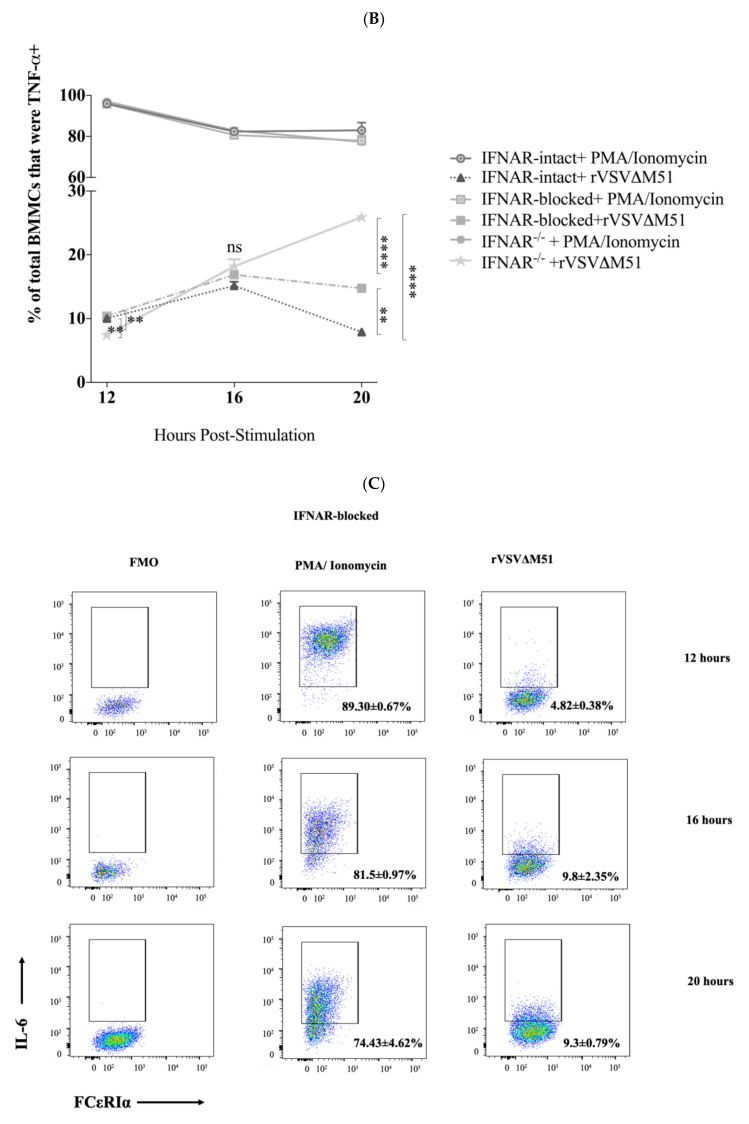

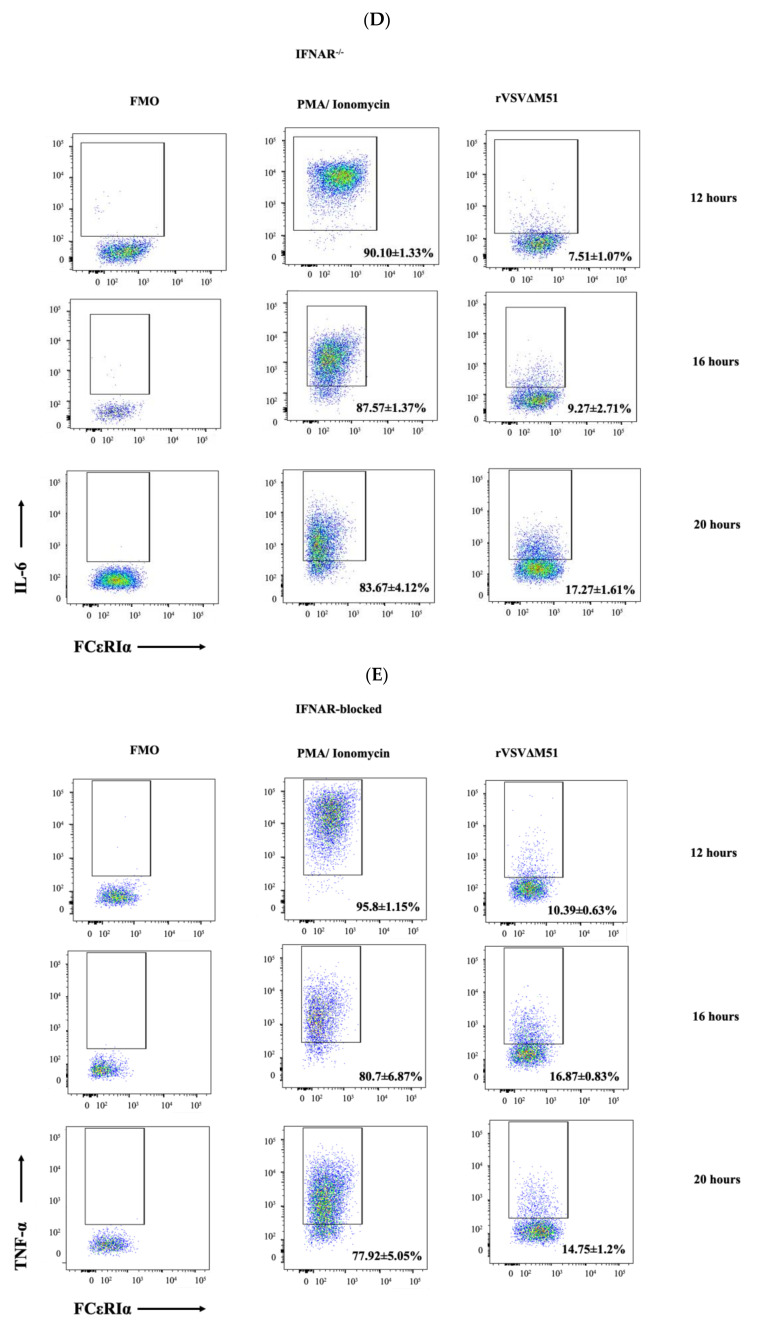

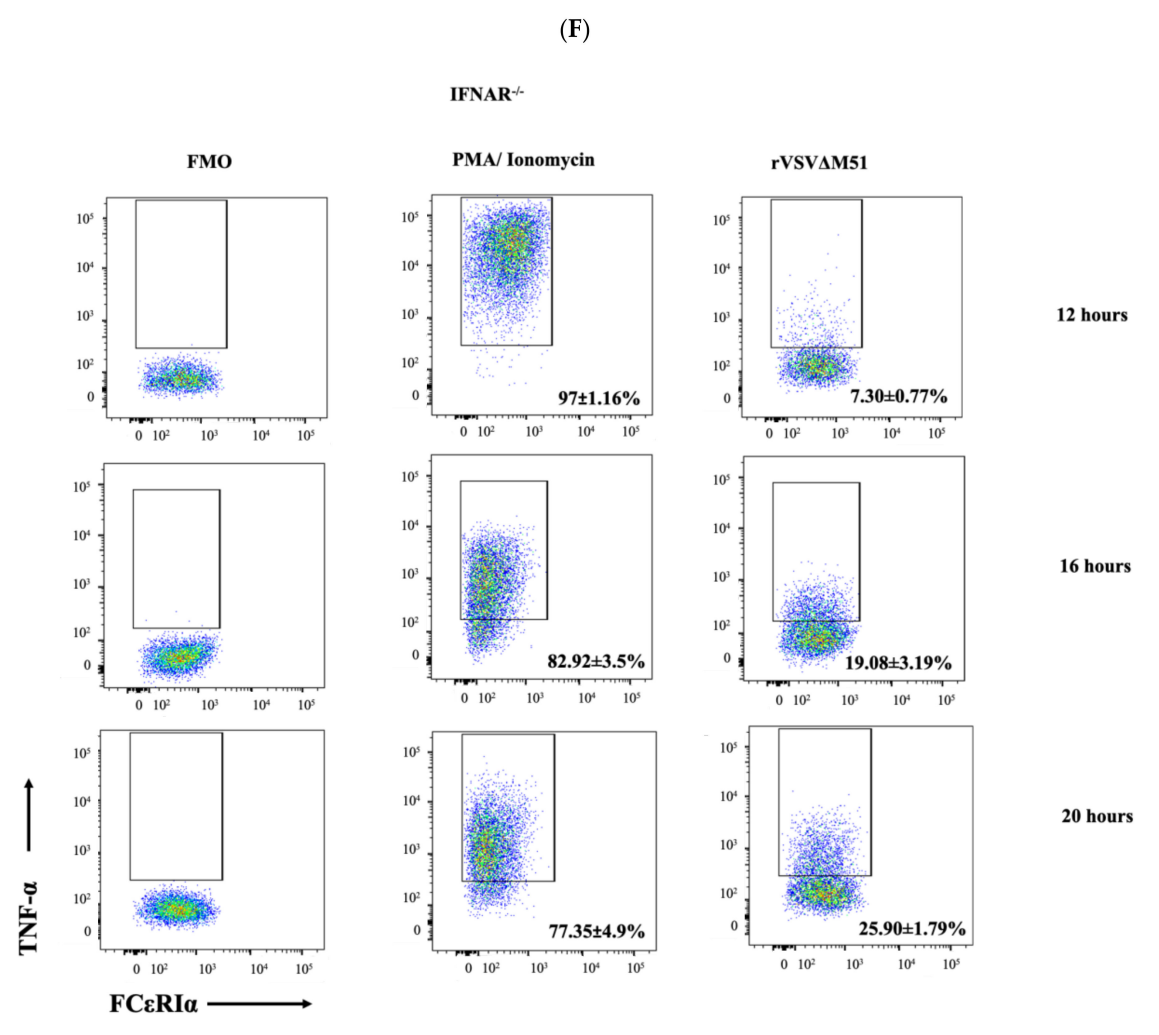
Recombinant vesicular stomatitis virus (rVSVΔm51)-induced cytokine production by murine bone-marrow-derived mast cells (BMMCs) was regulated by type I interferon receptor (IFNAR) signaling. BMMCs that were left untreated (intact BMMCs) or incubated with an antibody that blocked IFNARs (IFNAR-blocked BMMCs) or derived from IFNAR-knockout mice (IFNAR^−/−^ BMMCs) were infected with rVSVΔm51 at a multiplicity of infection of 10. After 12, 16, and 20 h, the cells were stained for the surface markers FcεRIα and c-kit, as well as intracellular cytokines interleukin (IL)-6 and tumor necrosis factor (TNF)-α. The cells were then analyzed by flow cytometry, and fluorescence minus one (FMO) controls were used to set gates to identify IL-6^+^ and TNF-α^+^ BMMCs. Expression of (**A**) IL-6 and (**B**) TNF-α expression in FcεRIα^+^c-Kit^+^ mast cells is shown after infection with rVSVΔm51 or treatment with phorbol 12-myristate 13-acetate (PMA) and ionomycin as a positive control stimulus. Graphs show means and standard deviations pooled from four (IFNAR-intact and -blocked BMMCs) or six (IFNAR^−/−^ BMMCs) experimental replicates. Two-way analysis of variance with Tukey’s multiple comparison test was used to define statistical significance as ** *p* < 0.001; *** *p* < 0.0005; **** *p* < 0.0001. Representative dot plots of (**C**) IL-6 expression in IFNAR-blocked and (**D**) IFNAR^−/−^ BMMCs as well as TNF-α expression in (**E**) IFNAR-blocked and (**F**) IFNAR^−/−^ BMMCs are shown.

To explore the potential effect of IFNAR signaling on antiviral cytokine responses of MCs, IFNAR-intact, IFNAR-blocked, and IFNAR^−/−^ BMMCs were treated with rVSVΔm51 at a MOI of 10 and assessed for cytokine production. PMA and ionomycin were used as control stimuli for detecting cytokines in BMMCs. All groups of cells showed an increase in production of cytokines between 12 and 16 h post-infection. However, there was a significant difference between BMMCs regarding IL-6 and TNF-α production at later time points. The frequency of BMMCs producing IL-6 and TNF-α decreased between 16 and 20 h post-infection in IFNAR-intact cells from 16.05% ± 2.51% and 15% ± 1.21% to 5.87% ± 0.64% and 7.92% ± 0.88%, respectively. In contrast, IFNAR^−/−^ BMMCs showed a steady increase in production of both cytokines between 16- and 20 h post-infection (17.27% ± 1.61% and 25.90% ± 1.79%, respectively). Although the production of both cytokines in IFNAR-blocked BMMCs was lower than that of IFNAR^−/−^ cells, it was still significantly higher than IFNAR-intact cells at 20 h post-infection (9.30% ± 0.79% for IL-6 and 14.75% ± 1.20% for TNF-α) (Figure 5). Together, these findings highlight the importance of IFNAR signaling in the ability of BMMCs to down-regulate antiviral cytokine responses after an infection. Specifically, disruption of type I IFN signaling during a viral infection can result in over-production of cytokines by MCs.

### 2.6. IFNAR Signaling Protected BMMCs from rVSVΔM51-Induced Cell Death

**Figure 6:**
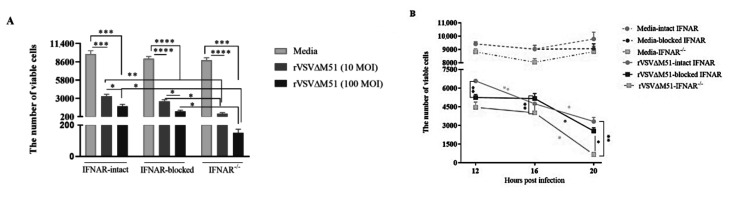
Type I interferon receptor (IFNAR) signaling protected murine bone-marrow-derived mast cells (BMMCs) from recombinant vesicular stomatitis virus (rVSVΔm51)-induced cell death. IFNAR-intact, IFNAR-blocked, and IFNAR-knockout (IFNAR^−/−^) BMMCs were infected with rVSVΔm51 at a multiplicity of infection (MOI) of 10 or 100. Twelve, 16, and 20 h later, the cells were stained for the surface markers FcεRIα and c-kit. A cell viability dye was used to discriminate live and dead BMMCs by flow cytometry. Graphs show means and standard deviations pooled from four (IFNAR-intact and IFNAR-blocked BMMCs) or six (IFNAR^−/−^ BMMCs) experimental replicates of the number of viable cells among FcεRIα^+^c-Kit^+^ mast cells at (**A**) 20 h post-infection with 10 or 100 MOI of the virus and (**B**) at 12, 16, and 20 h post-infection with 10 MOI of the virus. Two-way analysis of variance with Tukey’s multiple comparison test was used to define statistical significance as * *p* < 0.05; ** *p* < 0.001; *** *p* < 0.0005; **** *p* < 0.0001.

To explore the potential effect of IFNAR signaling on virus-induced cell death, IFNAR-intact, IFNAR-blocked, and IFNAR^−/−^ BMMCs were treated with medium or rVSVΔm51 at a MOI of 10. After 12, 16, and 20 h, the cells were stained with a fixable viability dye to look at the number of viable cells using flow cytometry. In contrast to medium-treated cells, all groups of virus-treated cells showed a reduction in cell viability after infection with rVSVΔm51 (Figure 6A). At 20 h post-infection, IFNAR^−/−^ BMMCs, which over-expressed cytokines (Figure 5), had a lower number of viable cells than IFNAR-blocked and -intact BMMCs (Figure 6B), both of which had relatively lower-magnitude cytokine responses (Figure 5) (651.75 ± 364.06 cells versus 2545.75 ± 520.85 cells versus 3316.25 ± 645.55 cells, respectively). There was no significant difference in viability of cells between IFNAR-intact and -blocked BMMCs at 20 h post-infection. IFNAR^−/−^ cells did not show a significant difference in the reduction of viable cells after 20 h post-infection with rVSVΔM51 at MOIs of 10 versus 100 (651.75 ± 364.06 cells versus 151 ± 45.32 cells, respectively) (Figure 6B). This suggested that virus-induced cytotoxicity was not dose-dependent in these cells. In contrast, the reduction in viability of IFNAR-blocked cells did correlate with the dose of the virus (2545.75 ± 520.85 cells versus 1035 ± 304.64 cells after treatment with rVSVΔM51 at MOIs of 10 and 100, respectively).

## 3. Discussion

In the current study it was observed that the magnitude of antiviral cytokine responses of MCs was controlled by IFNAR signaling and that IFNAR signaling protected MCs from virus-induced cell death. Indeed, BMMCs produced IL-6 and TNF-α in response to rVSVΔM51, and intact IFNAR signaling was required for shutting these responses down and preventing the cells from dying.

The prevalence of MCs at common points of entry for pathogens, as well as expression of a broad array of potential entry receptors, and a wide range of inflammatory mediators have made them ideal targets for a number of viruses. In the submucosa of the respiratory tract and in the nasal cavity, viruses are sensed by MCs and release early inflammatory compounds such as histamine and proteases followed by the generation of cytokines like IL-6 [46]. It is important to note that IL-6 can play a pro-inflammatory role early in the innate response but then an anti-inflammatory role at later time points [47]. The susceptibility of MCs to viral infection varies between viruses [24,25,26,28,44,48,49]. However, viral infection of MCs may not necessarily lead to the release of infectious virions [24,37,48]. While this study shows that BMMCs could be infected with VSV, whether the infection was productive is a question that future studies may address. However, it is important to note that leukocytes have generally been thought to be resistant to infection with VSV.

In vitro, MCs have been reported to produce inflammatory cytokines and chemokines in response to different stimuli, including lipopolysaccharides [50,51], synthetic viral double-stranded RNA, as well as infection of viruses [20,49] including VSV [27,52]. In this study, in vitro-cultured BMMCs were shown to produce the cytokines IL-6 and TNF-α in response to rVSVΔM51. We could not detect significant cytokine expression by BMMCs earlier than 12 h after infection with rVSVΔM51, suggesting that the cytokines were generated as a consequence of de novo synthesis rather than being released from pre-formed pools [53]. A modest but statistically significant release of IL-6 and TNF-α in response to rVSVΔM51 was observed at 12 h post-infection, which peaked by 16 h and declined by 20 h post-infection. Moreover, we demonstrated that while IFNAR signaling does not affect the amount of TNF-α produced by BMMCs in response to rVSVΔM51 at 16 h post-infection, IL-6 generation was higher in IFNAR-intact BMMCs compared to IFNAR-blocked and IFNAR^−/−^ BMMCs. This could be due to enhancement of IL-6 production by type I IFNs, which was previously reported in human neutrophils [54]. In such a scenario both IFNAR-blocked and IFNAR^−/−^ BMMCs failed to respond to type I IFNs to potentiate early IL-6 production. Interestingly, at a later time point where IFNAR signaling could shut down cytokine responses in IFNAR-intact BMMCs, IFNAR-blocked and IFNAR^−/−^ BMMCs continued to produce cytokines to toxic levels. Indeed, IFNAR signaling was required to control this cytokine production and protect the MCs from death. Relatively lower production of cytokines in IFNAR-blocked BMMCs than that of IFNAR^−/−^ cells may imply that the antibody-mediated blockade of IFNARs might have been incomplete and that some level of IFNAR signaling was retained during infection with rVSVΔm51. If true, this would support the importance of functional IFNAR signaling in the regulation of antiviral cytokine responses.

The recombinant VSVΔm51-induced cell death in BMMCs observed in this study aligns with previous reports on attenuated strains of VSV that were found to be highly lytic in IFN-non-responsive human tumor cell lines from the NCI-60 panel (leukemia, colon carcinoma, non-small-cell lung carcinoma, melanoma, ovarian carcinoma, renal carcinoma, and prostate and breast cancer), as compared with IFN-responsive cells [55,56]. Importantly, our findings highlight rVSVΔM51-induced cell death in primary cultures of MCs. This may be particularly relevant to oncolytic virotherapies that rely on rhabdoviruses, especially since these are versatile innate sentinel cells that can profoundly shape host immune responses to a viral infection. Infection and killing of MCs during rhabdovirus-mediated oncolytic virotherapies might be an unappreciated off-target effect especially for therapies that combine oncolytic rhabdoviruses with potent suppressors of type I interferon signaling [57]. Further, MCs might contribute to adverse events that are associated with exaggerated cytokine responses in some patients treated with oncolytic viruses. These concerns deserve thorough investigation in the future.

Moreover, influenza A virus-induced apoptosis in the P815 mouse mastocytoma cell line was associated with virus replication and production of pro-inflammatory cytokines and chemokines, including IL-6 and TNF-α. This virus-induced apoptosis was implicated in the pathogenesis of influenza A virus infections [49]. Apoptosis of MCs has also been reported to be induced by other viruses, including dengue virus [58] and rhinovirus [59]. Dengue virus-induced apoptosis in human MCs and MC lines was also associated with production of pro-inflammatory cytokines and chemokines [60]. This current study, however, did not evaluate the type of programmed cell death that in vitro-cultured MCs underwent during infection with rVSVΔm51.

Taken together, the results of this study emphasize the role of IFNAR signaling in controlling the magnitude of antiviral cytokine responses by MCs. Given that MCs are among the early cytokine-producing cells during viral infections [27] and given their well-established contributions to toxic cytokine [61] responses during severe pathogenic viral infections [62], it is important to define the early virus-induced events that lead to unbridled inflammatory responses by these versatile cells. According to the findings of this study, it is tempting to speculate that virus-induced type I IFN responses in epithelial and mucosal cells modulate tissue-resident MCs. In the event of viral invasion associated with impaired IFNAR signaling, unrestrained inflammatory responses of MCs could lead to excessive infiltration of inflammatory cells and subsequent generation of a cytokine storm, thereby causing the development of immune-mediated pathogenesis. Further understanding of IFNAR-mediated antiviral cytokine responses of MCs might facilitate the rational development of novel strategies to prevent or treat pathogenesis associated with excessive inflammatory responses by MCs during viral infections.

## 4. Materials and Methods

### 4.1. Mice

Six- to 8-week-old female C57BL/6 mice were purchased from Charles River Laboratories, Senneville, QC, Canada. IFNAR^−/−^ mice from the C57BL/6 background were kindly provided by Dr. Yonghong Wan (McMaster University, Hamilton, ON, Canada) with the permission of Dr. Laurel Lenz (University of Colorado, Denver, CO, USA) and were used as bone marrow donors for in vitro culture and development of BMMCs. All mice were kept in an isolated specific pathogen-free facility at the University of Guelph (Guelph, ON, Canada). Use of mice was approved by the University of Guelph Animal Care Committee (animal utilization protocol #3807) and adhered to the standards recommended by the Canadian Council on Animal Care.

### 4.2. Viruses

A replication-competent WT recombinant Indiana strain of VSV carrying a transgene encoding GFP and a highly attenuated version with a deletion at position 51 of the matrix protein to remove suppression of anti-viral type I IFN responses (rVSVΔm51) [63] were kindly provided by Dr. Brian Lichty, McMaster University. These were used to study antiviral cytokine responses of BMMCs. The viruses were used in containment level 2 facilities under the approval of the University of Guelph biosafety committee (biohazard permit #A-367-04-19-05).

### 4.3. In vitro Differentiation of BMMCs

BMMCs were generated according to a previously described method [64]. Femurs and tibias were harvested from mice, and the marrow was flushed aseptically with Hanks’ Balanced Salt Solution (Hyclone; cat #SH3058802, Ottawa, ON, Canada) using syringes with 23-gauge (BD; cat. #14-826-6B) and 18-gauge (BD; cat. #148265D) needles for flushing and mixing, respectively, followed by passing the homogenized single cell suspension through a 70 μm cell strainer (Fisher Scientific; cat. #22363548, Waltham, MA, USA) and then centrifuging at 500× *g* for five minutes. The resultant cell pellet was resuspended in a complete BMMC medium (RPMI-1640 medium (Hyclone; cat. #SH3002701, Ottawa, ON, Canada) supplemented with, 0.1 mmol/L MEM nonessential amino acid solution (Corning, cat. #MT25025CI, Waltham, MA, USA), 0.1 mmol/L sodium pyruvate (Gibco cat. #11360-070, Waltham, MA, USA), 2-mercaptoethanol (1:1000) (Life Technologies; cat. #21-985-023, Carlsbad, CA, USA), penicillin (100 units/mL), streptomycin (100 units/mL) (Hyclone; cat. #SV3001, Ottawa, ON, Canada) and 10% (*v*/*v*) fetal bovine serum (VWR Life Science Seradigm; cat. #97068-085, Mississauga, ON, Canada) and 20% (*v*/*v*) pokeweed mitogen-stimulated spleen cell-conditioned medium [PWM-SCM]) to give 1 × 10^5^ cells/mL in tissue-culture flasks incubated at 37 °C in a 5% CO_2_ humidified atmosphere. Cell culture media was refreshed once per week by centrifugation and resuspension of resulting cell pellets in fresh complete BMMC medium. It was shown previously that PWM-SCM differentiates bone-marrow-derived cells into a phenotype representative of pulmonary mast cells [65,66].

### 4.4. Preparation of PWM-SCM

PWM-SCM was prepared as described [67] with minor modifications. Specifically, 2 × 10^6^ splenocytes per mL of BMMC media were cultured from C57BL/6 mice and stimulated with 8 μg/mL of lectin (Sigma; cat. #L8777, Oakville, ON, Canada) for five days at 37 °C in a 5% CO_2_ humidified incubator. Afterwards, the medium was collected, centrifuged for 15 min at 3005× *g*, and filtered through a 0.2 micron filter and archived in small aliquots in a −20 °C freezer until being used for the development of mature BMMCs.

### 4.5. Characterization and Treatment of BMMCs

In vitro cultured BMMCs (1 × 10^5^ cells) were cytospun and stained with Wright–Giemsa for assessment of granularity via light microscopy. Purity of BMMCs was confirmed by flow cytometric analysis of the surface markers using FITC-conjugated anti-mouse CD117 (c-Kit) (clone 2B8, eBioscience; cat. #105806), APC/Cy7-conjugated anti-mouse FCεRIα (clone MAR-1, BioLegend; cat. #134326, San Diego, CA, USA), and PerCP/Cy5.5-conugated anti-mouse IL-33Rα (IL1RL1, ST2) (clone DIH9, BioLegend; cat. #145312, San Diego, CA, USA). Five- to 10-week-old BMMCs that were more than 95% double-positive for c-kit and FcεRI-α were used for experimental purposes.

Viable BMMCs were counted using 0.4% trypan blue dye (Gibco; cat. #15250, Waltham, MA, USA) exclusion, and 9 × 10^5^ cells were plated in fresh complete BMMC media in a flat-bottom 96-well plate and stimulated with a range of doses of rVSVΔm51. To detect intracellular IL-6 and TNF-α, the cells were incubated with brefeldin A (1:1000) (BD Biosciences; cat. #420601, San Jose, CA, USA) 10 h before the end of the experiment to retain cytokines within the cells. Stimulation of cells with 20 ng/mL of PMA (Sigma; cat. #P1585, Oakville, ON, Canada) and 500 ng/mL of ionomycin (Sigma; cat. #I9657, Oakville, ON, Canada) was used as a positive control for detection of intracellular IL-6 and TNF-α in BMMCs. To study the role of IFNAR signaling in cytokine responses of BMMCs to rVSVΔm51, WT BMMCs were treated with 10 μg/mL of anti-mouse IFNAR-1 (clone MAR1-5A3, Leinco Technologies; cat. #0516L270, Fenton, MO, USA) two hours before stimulation with rVSVΔm51 or PMA and ionomycin. Three groups of cells were used in these experiments: WT BMMCs with intact IFNAR signaling, WT BMMCs treated with anti-IFNAR1 to block IFNAR signaling, and IFNAR^−/−^ BMMCs without IFNAR gene expression.

### 4.6. Flow Cytometric Detection of Intracellular Cytokines in BMMCs

Stimulated BMMCs were transferred into a U-bottom 96-well plate to be stained for surface markers and intracellular cytokines for further analysis by flow cytometry. To that end, the cells were spun down at 500× *g* at 4 °C for five minutes, the supernatant was removed, and cells were resuspended in flow cytometry staining buffer containing phosphate-buffered saline (Hyclone; cat. #SH0325601, Ottawa, ON, Canada) and 0.1% bovine serum albumin (BD Biosciences; cat. #BP1600100, San Jose, CA, USA) containing anti-mouse CD16/32 (clone 93, BD Biosciences; cat. #101321, San Jose, CA, USA) for 15 min at 4 °C to block F_C_ receptors. The cells were then stained in flow cytometry staining buffer containing FITC-conjugated anti-mouse c-Kit, APC/Cy7-conjugated anti-mouse FCεRIα, and PerCP/Cy5.5-conjugated anti-mouse ST2. After a 20 min incubation at 4 °C in the dark, the cells were washed with flow cytometry staining buffer, stained with a fixable viability dye (Zombie Aqua; BioLegend, cat. #423101/423102, San Diego, CA, USA), and incubated at 4 °C in the dark for an additional 20 min. The BMMCs were washed twice with phosphate-buffered saline and resuspended in intracellular fixation buffer (BioLegend, cat. #420801, San Diego, CA, USA) for 30 min at room temperature in the dark. Afterwards, cells were washed once with intracellular staining permeabilization buffer (1X) (BioLegend; cat. #421002, San Diego, CA, USA) and resuspended in permeabilization buffer containing PE-conjugated anti-mouse TNF-α (clone MP6-XT22, BioLegend; cat. #506306, San Diego, CA, USA) and APC-conjugated anti-mouse IL-6 (clone MP5-20F3, BioLegend; cat. #504508, San Diego, CA, USA). After 50 min incubation at room temperature in the dark, the cells were washed with permeabilization buffer and resuspended again in permeabilization buffer and incubated at 4 °C in the dark for an extra 15 min. The cells were then spun down and resuspended in flow cytometry staining buffer and run on a FACS Canto II flow cytometer (Becton Dickinson) using FACS Diva Software Version 8 for data acquisition. The resultant data were then analyzed using FlowJo Software version 10 (FlowJo LLC, Ashland, OR, USA). The gating strategy used for flow cytometric analysis of BMMCs is shown in Supplementary Figure S1.

### 4.7. Flow Cytometric Detection of Viable BMMCs

Viable cells were counted using an inverted light microscope and 0.4% trypan blue dye (Gibco; cat. #15250, Waltham, MA, USA) exclusion, and 1 × 10^4^ cells were plated in fresh complete BMMC media containing 20% PWM-SCM in a flat-bottom 96-well plate and stimulated with a range of doses of rVSVΔm51. At the indicated time, BMMCs were stained for mast cell surface markers prior to staining with a fixable viability dye (Zombie Aqua; BioLegend, cat. #423101, San Diego, CA, USA). Ninety percent of all the cells in all samples were run through a BDCanto II flow cytometer for data acquisition. BMMCs negative for Zombie Aqua were considered as viable cells.

### 4.8. Time-Lapse Images of VSV-Infected BMMCs

BMMCs were infected with WT VSV expressing a GFP transgene at a MOI of 10 in a 25 mm cell culture Petri dish, and real-time images were recorded over the course of 24 h using time-lapse microscopy (LumaScope, Bioimager). Representative images were extracted from the files that were generated.

### 4.9. Statistics

GraphPad Prism version 8, GraphPad Software, San Diego, CA, USA was used for all graphing and statistical analyses. Graphs show means and standard errors. One- or two-way analysis of variance was used when means of more than two independent groups were subject to comparison across one or more time points, respectively. Statistical significance was defined as *p*-values < 0.05. Student’s *t*-tests were used when there was one variable with two groups (Figure 1).

## Supplementary Materials

Supplementary materials can be found at https://www.mdpi.com/1422-0067/21/23/9041/s1.

## Abbreviations

BMMC	bone-marrow-derived mast cell
GFP	enhanced green fluorescent protein
IFNAR	type I interferon receptor
IFN	interferon
IL	interleukin
MC	mast cell
PWM-SCM	pokeweed mitogen-stimulated spleen cell-conditioned medium
rVSVΔm51	recombinant vesicular stomatitis virus with a deletion of methionine at amino acid position 51
TNF	tumor necrosis factor
TLR	Toll-like receptor
WT rVSV	wild-type recombinant vesicular stomatitis virus (lacking the Δm51 mutation)
