# Supplementary material for: Type I Interferon α/β Receptor-Mediated Signaling Negatively Regulates Antiviral Cytokine Responses in Murine Bone-Marrow-Derived Mast Cells and Protects the Cells from Virus-Induced Cell Death
Source: Int J Mol Sci. 2020 Nov 27;21(23):9041. doi: 10.3390/ijms21239041 (PMC7729593; doi:10.3390/ijms21239041)
Supplement: Supplementary file 1 [file ijms-21-09041-s001.pdf]

A

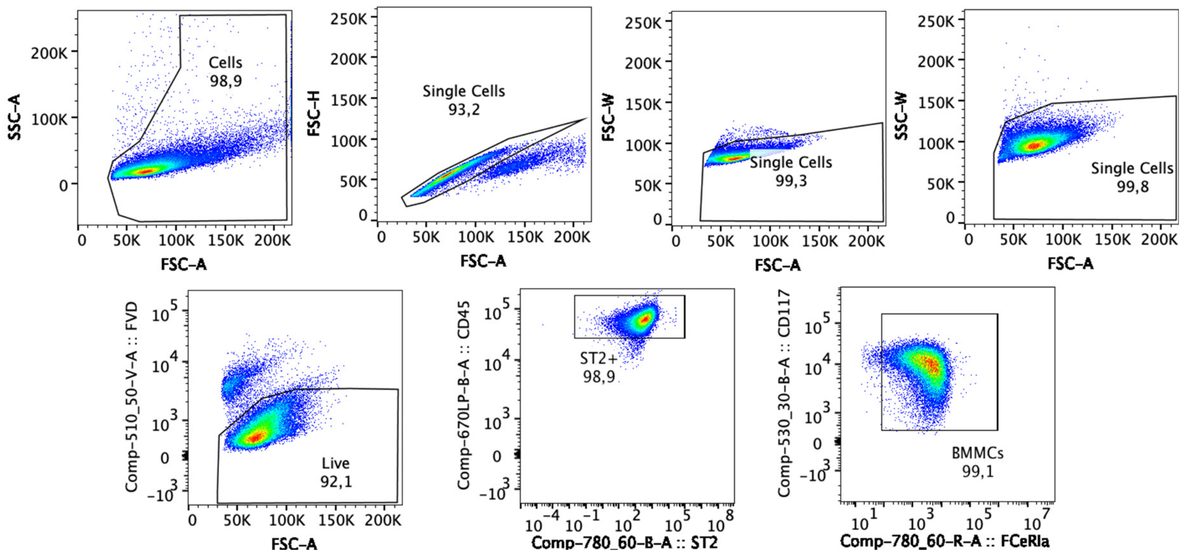

B

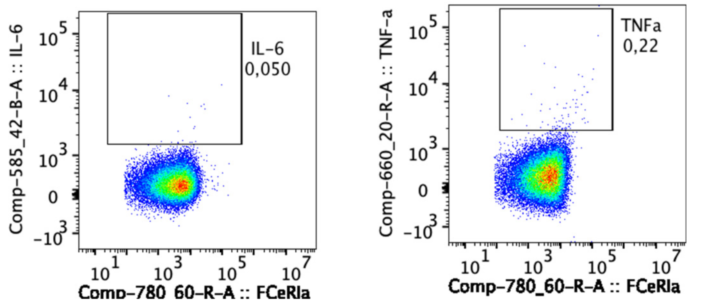

C

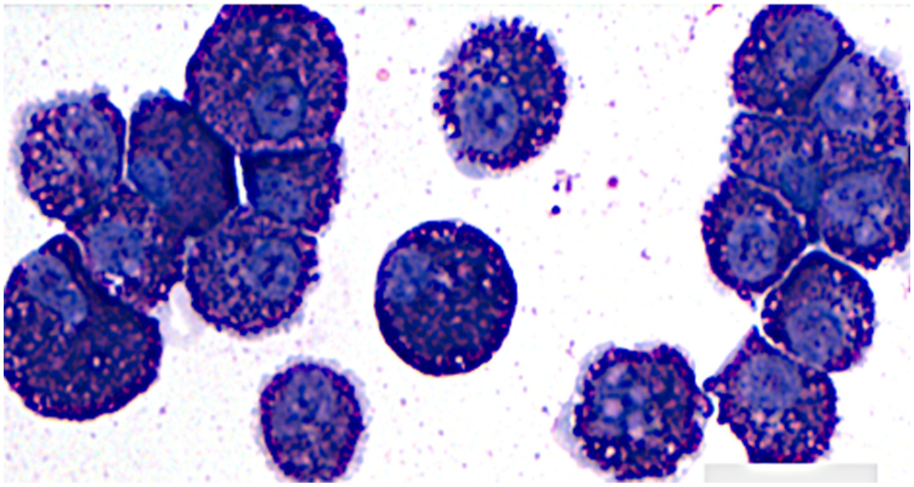

Supplementary Figure 1. Mouse bone marrow-derived cells cultured in the presence of pokeweed mitogen-stimulated spleen cell-conditioned medium (PWM-SCM) exhibited features of mast cells. Bone marrow-derived cells were obtained from three-week-old female C57BL/6 mice and cultured in 20% (V/V) PWM-SCM-containing media. After three weeks, the purity of bone marrow-derived mast cells was assessed using flow cytometry by staining them with surface-staining antibodies against FcεRIα, CD117 (c-kit), and IL-33 receptor ST2. (A) A side Scatter (SSC) / forward Scatter (FSC) gate was initially applied to exclude electronic noise and cell debris,

followed by singlet gates to exclude doublets, then a viability gate to exclude dead cells. Cells were defined as mast cells based on positive staining for the following markers: CD45, ST2, FcεRIα and CD117. (B) Intracellular cytokine staining and flow cytometry analysis revealed that the mast cells failed to express substantial quantities of the cytokines interleukin-6 and tumor necrosis factor-α in the absence inflammation. (C) Cell granularity was assessed using Wright-Giemsa staining of a cytospin prepared from 1×10^5 cells.
